# Region-Specific Responses of Adductor Longus Muscle to Gravitational Load-Dependent Activity in Wistar Hannover Rats

**DOI:** 10.1371/journal.pone.0021044

**Published:** 2011-06-22

**Authors:** Takashi Ohira, Masahiro Terada, Fuminori Kawano, Naoya Nakai, Akihiko Ogura, Yoshinobu Ohira

**Affiliations:** 1 Graduate School of Frontier Biosciences, Osaka University, Toyonaka City, Osaka, Japan; 2 Graduate School of Medicine, Osaka University, Toyonaka City, Osaka, Japan; McMaster University, Canada

## Abstract

Response of adductor longus (AL) muscle to gravitational unloading and reloading was studied. Male Wistar Hannover rats (5-wk old) were hindlimb-unloaded for 16 days with or without 16-day ambulation recovery. The electromyogram (EMG) activity in AL decreased after acute unloading, but that in the rostral region was even elevated during continuous unloading. The EMG levels in the caudal region gradually increased up to 6th day, but decreased again. Approximately 97% of fibers in the caudal region were pure type I at the beginning of experiment. Mean percentage of type I fibers in the rostral region was 61% and that of type I+II and II fiber was 14 and 25%, respectively. The percent type I fibers decreased and *de novo* appearance of type I+II was noted after unloading. But the fiber phenotype in caudal, not rostral and middle, region was normalized after 16-day ambulation. Pronounced atrophy after unloading and re-growth following ambulation was noted in type I fibers of the caudal region. Sarcomere length in the caudal region was passively shortened during unloading, but that in the rostral region was unchanged or even stretched slightly. Growth-associated increase of myonuclear number seen in the caudal region of control rats was inhibited by unloading. Number of mitotic active satellite cells decreased after unloading only in the caudal region. It was indicated that the responses of fiber properties in AL to unloading and reloading were closely related to the region-specific neural and mechanical activities, being the caudal region more responsive.

## Introduction

Removal of weight-bearing activity induces a remarkable effect on the muscles responsible for maintenance of posture and ground support, i.e., muscles composed predominantly of slow-twitch fibers [1]–[7]. Pronounced atrophy and shift toward a faster fiber phenotype, particularly in slow-twitch soleus muscle, has been a consistent finding in response to chronic gravitational unloading of human and rats. One of the causes for the unloading-related changes in muscle properties is often attributed to a decrease in neuromuscular activation [4], [5], [8]–[10]. The daily activation levels (as reflected by chronic recording of electromyogram, EMG) of rat soleus decrease in response to hindlimb unloading, although these levels gradually increase and return to the normal levels within 1 to 2 weeks of unloading [5], [9], [10]. Such responses of EMG levels were also closely associated with the afferent neurogram [9], [10]. Further, it was reported that 14 days of hindlimb unloading caused a reduction of the threshold and maximal intensity of the neurographic analogue of the H-reflex induced by the soleus afferent excitation in rats [11]. According to the study of Desaphy et al [12], the responses of fiber size and myosin heavy chain (MHC) expression of rat soleus to unloading and reloading were closely associated with the reversible changes of resting sarcolemmal chloride conductance. However, the soleus continue to atrophy in spite of the return to normal activation levels [5], [8], [13], clearly indicating that the direct cause of atrophy is not simply a decrease in the activation level of muscle.

In addition to changes in activation associated with unloading, there are also decreases in active and passive force development. During unloading, the ankle joints are chronically extended, resulting in a passive shortening of the ankle plantarflexors [4], [10], [14]. In this “slack” position, force production is minimized further, even if the muscles are activated. For example, Kawano et al. [10] reported that the tension development at the distal tendon of the soleus muscle was minimal because of the plantarflexion of ankle joints, maintained at ∼140 to 160°, during hindlimb suspension of rats. Thus, both the active and passive tension levels of the plantarflexors would be expected to be relatively low, particularly during the early phases (up to 2 weeks) after the initiation of unloading [10]. All of these data strongly indicate that the muscle adaptations induced by gravitational unloading are directly associated with the levels of loading and/or activation.

The similar changes, observed in soleus muscle [15], were also induced in the adductor longus (AL) muscle following gravitational unloading [16]. However, the precise mechanism responsible for the adaptation of AL muscle of rat to unloading is still unclear. Therefore, the current study was performed in order to test the hypothesis that the properties of AL muscle fibers, such as fiber cross-sectional area (CSA) and phenotype and/or distribution of satellite cells and myonuclei in fibers, are regulated by both neural and mechanical stimuli, which are influenced by gravitational unloading and loading.

## Materials and Methods

### Experimental design and animal care

All experimental procedures were conducted in accordance with the *Guide for the Care and Use of Laboratory Animals* of the Japanese Physiological Society. This study was also approved by the Committee on Animal Care and Use at Osaka University and Japan Aerospace Exploration Agency. All surgeries were performed under aseptic conditions with the rats under deep anesthesia (sodium pentobarbital, 5 mg/100 g body weight, *i.p.*).

A total of 25 male Wistar Hannover rats (Nihon CLEA, Tokyo) with the age of 5 weeks old and body weight of 150±3 g (Mean±SEM) was used. The rats were randomly separated into the cage-control (n = 15) and hindlimb-unloaded (n = 10) groups (Table 1). Five control rats were killed immediately before the initiation of experiment as the pre-experimental control. A single injection of 5-bromo-2′-deoxyuridine (BrdU, Sigma, 100 mg/kg body weight) in phosphate-buffered saline (PBS) was performed *i.p.* 2 days prior to the sampling. The amount of solid diet (CE-2, Nihon CLEA, Tokyo), which was completely eaten within ∼12 hr, was supplied at ∼10 a.m. daily (∼20 g/day/rat). Temperature and humidity in the animal room with 12∶12 hr light:dark cycle were maintained at ∼23°C and ∼55%, respectively.

**Table 1:** Changes in body weight (BW) and absolute and relative weight of adductor longus (AL) muscle.

Body weight	Pre	Day 16	Day 32
Control (g)	150±3	256±3*	330±6*, §
Unloaded (g)	―	222±6*, †	293±5*, †, §
AL weight	Pre	Day 16	Day 32
Control (mg)	33.5±2.1	55.6±2.5*	71.2±2.1*, §
(% BW×10-3)	22.3±1.1	21.7±0.9	21.6±0.5
Unloaded (mg)	―	29.6±2.5†	60.8±4.3*, §
(% BW×10-3)	―	13.4±1.4*, †	20.8±1.2§

Mean±SEM.

*, †, and §: p<0.05 vs. pre, age-matched control, and respective group at Day 16, respectively. Pre, Day 16, and Day 32: before hindlimb unloading, immediately after termination of 16-day unloading or cage housing, and 16 days after ambulation recovery or 32-day cage housing, respectively. n = 5 in each group/stage.

Gravitational unloading of hindlimbs was performed in the experimental group, as was described previously [4], [5], [10], [17], [18]. After 16 days, both control and unloaded rats (n = 5 for each group) were killed under anesthesia by *i.p.* injection of sodium pentobarbital (5 mg/100 g body weight). The hindlimb-unloaded rats were anesthetized while the hindlimbs remained unloaded to avoid any effects of acute loading. Following the body weight measurement, the right AL muscle was sampled for cross-sectional histochemical and/or immunohistochemical analyses. After measurement of wet weight, the muscles were gently stretched to a near optimum length *in vivo*, pinned on a cork, and frozen in isopentane cooled with liquid nitrogen. And the mid-belly region of muscle was mounted perpendicularly on a cork using OCT (optimum cutting temperature) compound (Miles, Elkhart, IN), quickly frozen in liquid nitrogen, and then stored at −80°C.

For the longitudinal analyses of the distribution of myonuclei and satellite cells in single fibers, the left muscles were sampled with a part of bony pelvis in order to save the fiber end. And the muscle was incubated in Dulbecco's modified Eagle's medium containing 0.2% type I collagenase, 1% antibiotics and 10% newborn calf serum for 4 hr at 35°C immediately to digest the collagens. Entire single muscle fibers in the rostral and caudal regions of the muscle were isolated from tendon to tendon (at least 30 fibers in each region) by using fine forceps under the microscope. Isolated fibers were immediately fixed with 4% paraformaldehyde at room temperature for 15 min.

After the termination of suspension, 5 rats in both control and unloaded groups were allowed to recover in the cages. These rats were killed and the AL muscles were sampled bilaterally and stored similarly following 16 days of ambulation recovery.

### Cross-sectional analyses of muscle fibers

#### i) Immunohistochemical staining

Standard immunohistochemical staining was performed in the serial cross-sections cut at 10 µm thickness in a cryostat maintained at −20°C. Muscle sections were fixed with 4% paraformaldehyde for 15 min. Blocking was performed using 10% donkey serum (Sigma, USA) in 1% triton X-100 in 0.1M PBS for 1 hr. The expression of MHC in individual fibers was analyzed by using mouse monoclonal antibodies specific to slow (type I) or fast (type II) MHC isoforms, i.e., primary antibody, NCL-MHCs and NCL-MHCf (Novocastra Laboratories, UK), as described previously [17], [19]. In addition, laminin was stained by using rabbit anti-laminin antibodies (Sigma, USA) to measure the fiber CSA. Primary antibodies were diluted 1∶100 with 0.1 M PBS containing 5% donkey serum and 0.3% triton X-100 and reacted with muscle sections for ∼2 days at 4°C. Alexa fluor 594 donkey anti-mouse IgG antibody and Alexa fluor 488 donkey anti-rabbit IgG antibody (Invitrogen, Japan) were used as the second antibodies. Second antibodies were diluted 1∶200 with 0.1 M PBS containing 5% donkey serum and 0.1% triton X-100 and reacted with muscle sections overnight at 4 °C. Finally, muscle sections were mounted by using medium, which contained hoechst 33342. Between each step, muscle sections were washed by immersing in 0.1M PBS for 5–20 min twice.

The stained images were detected by a confocal laser scanning microscopy and then incorporated into a computer. Numbers of primary antibody positive fibers were counted in slow and fast MHC-stained cross sections. Fiber phenotypes were classified as type I, I+II, or II and fiber CSA was analyzed by image processing software, Scion image (Scion corp.). Approximately 100 fibers were analyzed in the rostral, middle, and caudal region of each muscle sample.

#### ii) Enzyme activities

Activities of mitochondrial enzyme, succinate dehydrogenase (SDH), and glycolytic enzyme, α-glycerophosphate dehydrogenase (GPD), were determined as was described elsewhere [20], [21]. The detailed procedures were explained as the supplemental Materials S1.

### Longitudinal analyses of muscle fibers

#### i) Immunohistochemical staining for myonuclei and satellite cells

The numbers of myonuclei and both total and mitotic active muscle satellite cells were analyzed by using immunohistochemical staining. Fifteen fibers from each region of AL were reacted with anti-human/mouse/rat chicken Pax7 antibody (R&D Systems, Inc., USA) to stain the total muscle satellite cells. Another 15 fibers were treated with 1 N hydrochloric acid at 37°C for 30 min and incubated with purified anti-BrdU antibody (BD Pharmingen, USA) to label the mitotic active muscle satellite cells. Alexa fluor 594 donkey anti-mouse IgG antibody was used as the second antibody. And the stained fibers were mounted by using the medium, which contained hoechst 33342, for analysis of myonuclear number.

#### ii) Confocal laser scanning microscopy

The stained images were detected by a confocal laser scanning microscopy (Olympus, Japan). A FV-300 confocal microscope with an argon laser (488 nm of peak wavelength) and a He-Ne laser (543 nm of peak wavelength) was used to analyze the fiber length, fiber CSA, and sarcomere length.

The number of hoechst-labeled nuclei was counted as myonuclei in 3 different regions of each single fiber. Then, the total number of myonuclei in a single muscle fiber was calculated using the average number of myonuclei in these areas and the lengths of entire muscle fiber. And the numbers of total (Pax7-positive) and mitotic active (BrdU-positive) muscle satellite cells were also counted. The lengths of entire fiber and 10 sarcomeres in the 3 regions of muscle were measured by Nomarski optic scanning techniques. And the mean sarcomere length was calculated. Fiber CSAs were also measured. The fiber CSAs were normalized as "measured fiber CSA × 0.0128 × the mean sarcomere length × 10 + 0.68", when the sarcomere length was 2.5 µm. Myonuclear domain size was calculated as (average fiber CSA × fiber length)/number of myonuclei per fiber.

### Estimation of neural activity and mechanical load in muscle fibers

#### i) Recording of EMG

Responses of EMG activities in AL muscle to gravitational unloading were determined in 5 male Wistar Hannover rats (5 weeks old). Detailed procedures for electrode implantation have been published elsewhere [5], [8], [17], [22] and the methods were explained as the supplemental Materials S1. Time course changes in the EMG activities in conscious rats on the floor and during unloading were determined. The EMG recordings were performed for approximately 1 hr during the light period (9–11 am) before suspension (at rest on the floor) and during the 16 days of unloading and ambulation recovery. No weight bearing was permitted throughout the experimental period during hindlimb unloading.

#### ii) Data analyses

The recordings of EMG were performed. The raw signals of EMG were sampled at 0.01 seconds of time constant and the bandwidth between 0 and 3 kHz, and amplified (×1000) using AB-621G (Nihon Kohden, Tokyo) with 5 MΩ of input impedance and 60 dB of common mode rejection ratio. The amplified raw signals were processed by a PowerLab/16sp (ML795, AD Instruments, Australia), an analog-to-digital (A/D) converter, digitized at 2 kHz per channel, and stored on a computer. The total integrated areas of EMGs were determined using a computer software package (Chart v4.0.1, AD Instruments Inc., Australia).

#### iii) Analyses of joint angles and the length of sarcomeres

The responses of hip, knee, and ankle joints to acute unloading were analyzed in 5 rats by taking pictures. These angles were compared with those during a sedentary quadrupedal prone position on the floor. The changes of the mean length of sarcomeres of AL were also estimated in these rats, as reported previously [10], [23]. The rat was sacrificed by over-dose injection of sodium pentobarbital (15 mg/100 g body weight) and AL muscles were exposed. The rat was placed on a styrene foam board and the hip, knee, and ankle joints in each limb were fixed at the specific angles equivalent to those during unloading or a sedentary quadrupedal prone position on the floor, bilaterally. The AL muscles were covered with absorbent cottons containing 4% buffered-formaldehyde and were removed after 2 hrs of fixation. Subsequently, each muscle was sampled and the structure of muscle was analyzed by measuring the lengths of outer three sides.

The muscle was then splitted into the rostral, middle, and caudal regions longitudinally and whole fibers were isolated from tendon-to-tendon (at least 30 fibers in each region) using fine forceps under the microscope. The fibers were mounted on a slide glass with coverslip with “struts” of hardened nail polish on the corners to minimize fiber compression. The length of 10 consecutive sarcomeres was measured in 3 different regions. Then, the average sarcomere length in a single muscle fiber was calculated.

### Statistical analyses

All data were presented as means±SEM. Statistical significance was examined by two or three-way analysis of variance followed by Scheffé's post hoc test. Differences were considered significant at the 0.05 level of confidence.

## Results

### Body weight

Changes of body weight in response to growth and/or hindlimb unloading are shown in Table 1. The mean weight in the cage control group increased ∼71% and ∼29% every 16-day experimental period, respectively (p<0.05). Although the weight in the experimental group also increased during the 16 days of unloading (∼48%, p<0.05), the growth rate was significantly less relative to that in the control group (−23%, p<0.05). However, the body weight of the previously unloaded group increased toward the age-matched control group after 16 days of ambulation (32%, p<0.05).

### Muscle weight

The absolute wet weight of AL in the control rats increased ∼66% and 28% every 16-day experimental period, respectively (Table 1, p<0.05). The growth-related increase of the weight in the experimental group was inhibited by unloading. The mean weight was significantly less than the age-matched control group (−47%, p<0.05). However, the weight was significantly elevated to the control level after 16-day recovery (105%, p<0.05). The weight relative to body weight in the control group remained stable. But that in the unloaded group decreased (8.9% vs. pre-experimental level, p<0.05). And 16 days of ambulation on the floor allowed the muscle weight to recover to the control level (7.4%, p<0.05).

### Fiber size

**Figure 1:**
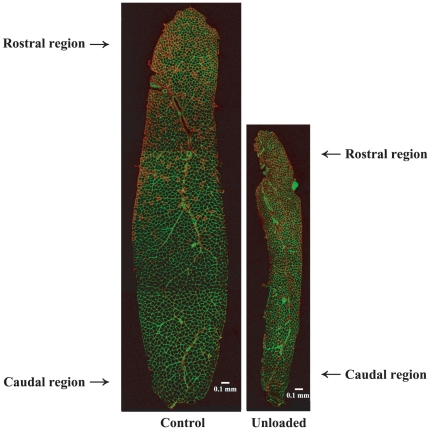
Mid-belly regions of adductor longus muscles sampled after 16-day unloading or cage housing. Cross-sections were stained immnohistochemically for fast myosin heavy chain. Red fibers are fast-twitch (type II or I+II) fibers.

**Figure 2:**
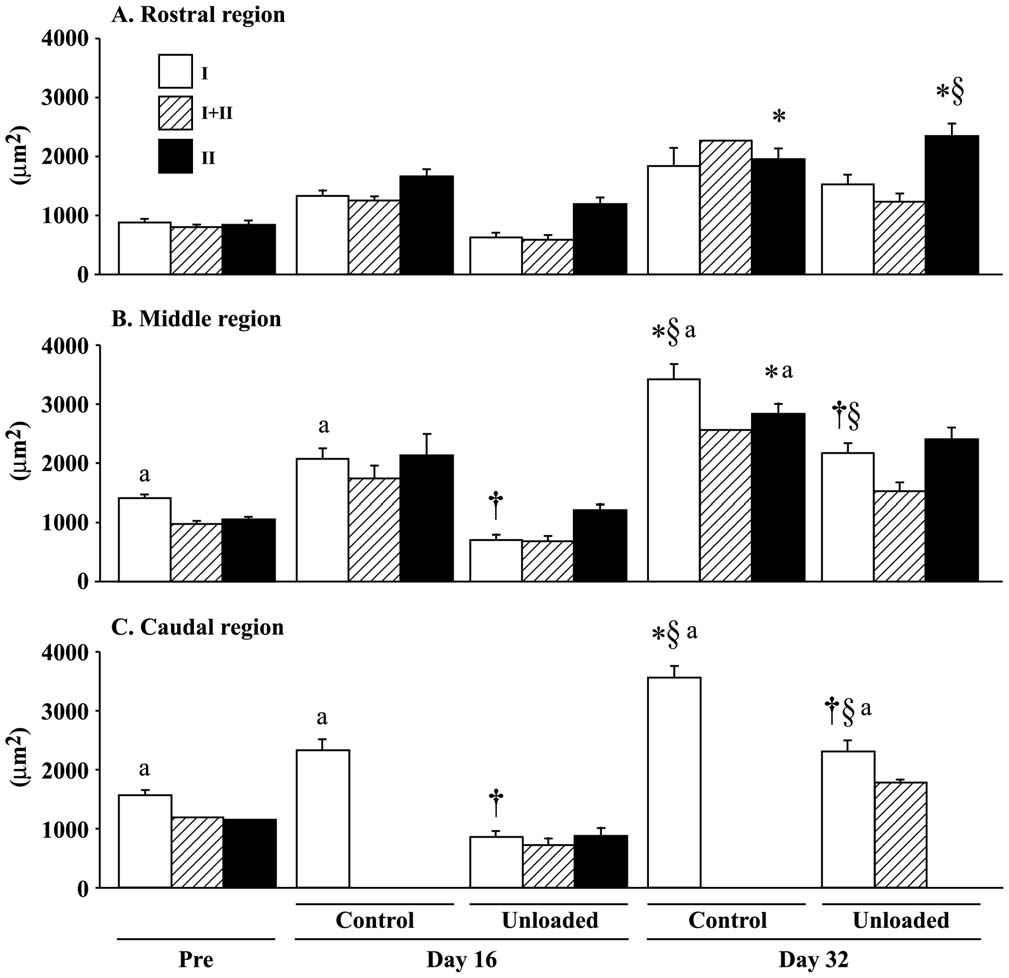
Region-specific responses of fiber cross-sectional area in adductor longus muscle. Mean±SEM. n = 5 in each group/stage. Pre, Day 16, and Day 32: Before hindlimb unloading, immediately after termination of 16-day unloading or cage housing, and 16 days after ambulation on the floor or 32 days of cage housing, respectively. I, II, and I+II: Fibers expressing pure type I (slow) and II (fast), and co-expressing both type I and II myosin heavy chain. *, †, and §: p<0.05 vs. pre, age-matched control, and respective group at Day 16, respectively. a: p<0.05 vs. the rostral region.

The cross-sectional images of whole AL muscles from the control and unloaded groups are shown in Figure 1. Muscle size in the unloaded group was clearly smaller than control. The fiber size in the caudal and middle regions was generally greater than that in the rostral region (Fig. 2). The mean fiber CSA in the control group gradually increased following growth. That of type II fibers in the rostral region, type I and II fibers in the middle region, and type I fibers in the caudal region significantly increased during 32 days of experiment (vs. pre-experimental level). Fiber CSA in the experimental group generally decreased after unloading, although muscle fiber size in the rostral region did not change significantly by unloading (−54% vs. age-matched control, p>0.05). The mean CSA of type I fibers in the middle (−66%) and caudal regions (−64%) at the end of unloading was significantly less than the age-matched control. After 16 days of ambulation recovery, that of type II fibers in the rostral region increased significantly. Further, that of type I fibers in the middle and caudal regions also increased, although the level was still less than the age-matched control (p<0.05).

### Fiber phenotype

**Figure 3:**
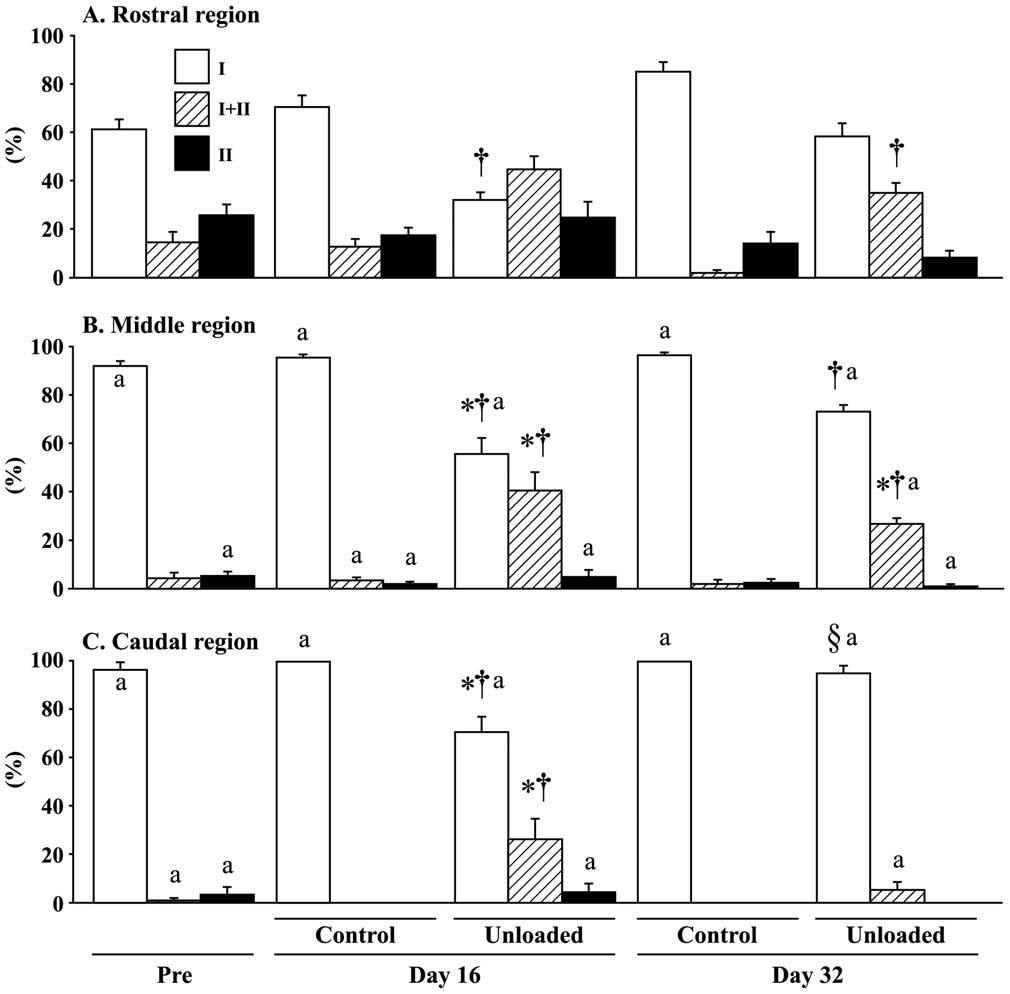
Region-specific responses of fiber phenotypes in adductor longus muscle. Mean±SEM. n = 5 in each group/stage. See Figure 2 for the symbols and abbreviations.

The typical distributions of fiber phenotypes in the control and unloaded groups are shown in Figure 1. Although AL muscle is composed of mainly slow-twitch fibers, the percent distribution of slow fibers was greater in the caudal and middle regions than the rostral region generally (Figs. 1 and 3). The caudal and middle regions of AL muscle in the control rats were composed of approximately 97 and 92% of fibers expressing pure type I MHC at the beginning of experiment, respectively (Fig. 3). Meanwhile, the mean percentage of pure type I fibers in the rostral region was 61% and the distribution of type I+II and II fibers was 14 and 25%, respectively.

After 16 days of growth on the floor, all fibers in the caudal region became pure type I (+3.5%, p>0.05, Fig. 3). The distributions of type I fibers in the rostral and middle regions also increased insignificantly (9.6 and 3.6%, respectively). The percentage of type I fibers in the middle and caudal regions in the unloaded group decreased from the pre-experimental levels (36.6, and 26.2%, p<0.05) and *de novo* appearance of type I+II fibers was noted. That in the rostral region also decreased (29.5%). However, according to the statistical analyses including type I, I+II, and II fibers, the decrease was not significant, although the mean change was greater than that in the caudal region (p>0.05). But change was significant, when the analyses were performed using type I fibers only. The percent type I fibers in all regions of the unloaded group was less than the age-matched control (39.1, 40.1, and 29.7% in the rostral, middle, and caudal regions, respectively, p<0.05). The percentages of type I+II fibers of unloaded group in the middle and caudal, not rostral (p = 0.054), regions were significantly greater than that of the pre-suspension and age-matched control levels (p<0.05). The fiber phenotypes in the caudal region were normalized after 16-day ambulation. But the unloading-related transformation toward fast type in the rostral and middle region was not normalized completely, although there was a trend to recover toward the control level. The percentage of type I and I+II fiber in the middle region and that of type I+II fiber in the rostral region were still different from those of the age-matched controls (p<0.05).

### Enzyme activity

Prominent fiber-type-specific differences in the enzyme activities were not observed, as was reported elsewhere [21]. Responses of the fiber-type-specific SDH and GPD activities (ΔOD/min) in whole muscle fibers to unloading were not prominent, either (Supplemental Materials S1, Figs. S1 and S3). The integrated activities of both SDH and GPD (ΔOD/min×fiber CSA) in whole muscle fibers tended to decrease after unloading and increase following ambulation recovery (p>0.05) (Supplemental Materials S1, Figs. S2 and S4).

### Distribution of myonuclei and satellite cells

**Figure 4:**
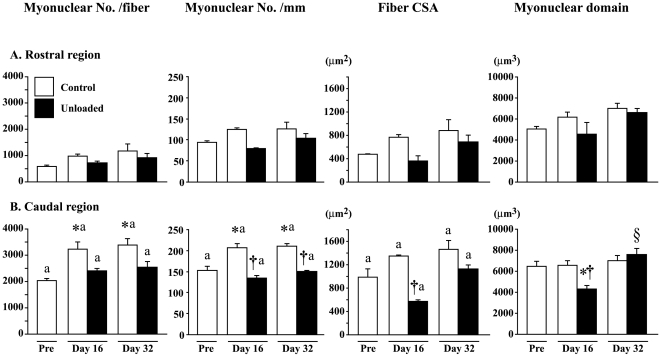
Myonuclear number per fiber and mm fiber length, fiber cross-sectional area (CSA), and myonuclear domain. Mean±SEM. n = 5 in each group/stage. See Figure 2 for the symbols and abbreviations.

The fiber length was longer in the caudal than the rostral region (data not shown). The number of myonuclei per fiber and per mm of fiber length was greater in the caudal than the rostral region (Fig. 4). Growth-associated increase of myonuclear number was noted especially in the caudal region of control rats. The increase during the first 16 days was significant in the caudal, not rostral, region. Growth-related increase of myonuclear number was inhibited by unloading. The distribution of myonuclei remained low even after 16-day ambulation recovery. The CSAs in the combined fibers were significantly greater in the caudal than the rostral region. The mean fiber CSA especially in the caudal region was decreased by unloading. That in the caudal region was significantly less than the age-matched control, but returned toward the control level after 16 days of recovery (p>0.05). Myonuclear domain size in the caudal, not rostral, region was also decreased by unloading. But, the unloading-related decrease of myonuclear domain was significantly increased after 16-day recovery.

**Figure 5:**
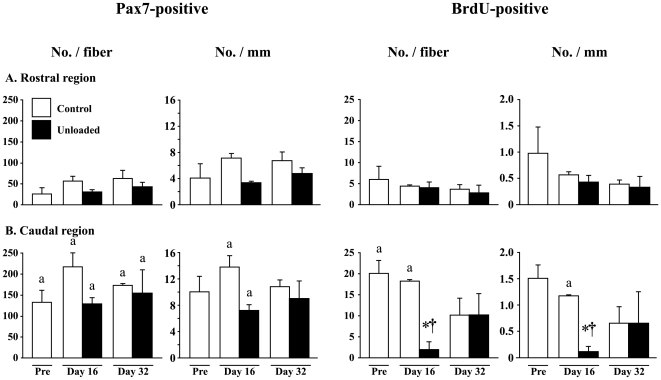
Satellite cell number per entire single fiber and mm fiber length. Responses of both total (Pax7-positive) and mitotic active (BrdU-positive) satellite cells are shown. Mean±SEM. n = 5 in each group/stage. BrdU: 5-bromo-2′-deoxyuridine. See Figure 2 for other symbols and abbreviations.

More number of satellite cells was distributed in the caudal than the rostral region (Fig. 5). The change of total number of muscle satellite cells (Pax7-positive) per fiber and per mm fiber length was not prominent, even though the growth-associated increase tended to be inhibited by unloading. The number of mitotic active (BrdU-positive) satellite cell per fiber and mm of fiber length decreased after unloading only in the caudal region, but returned to the control level after 16 days of ambulation.

### Electromyogram activity

**Figure 6:**
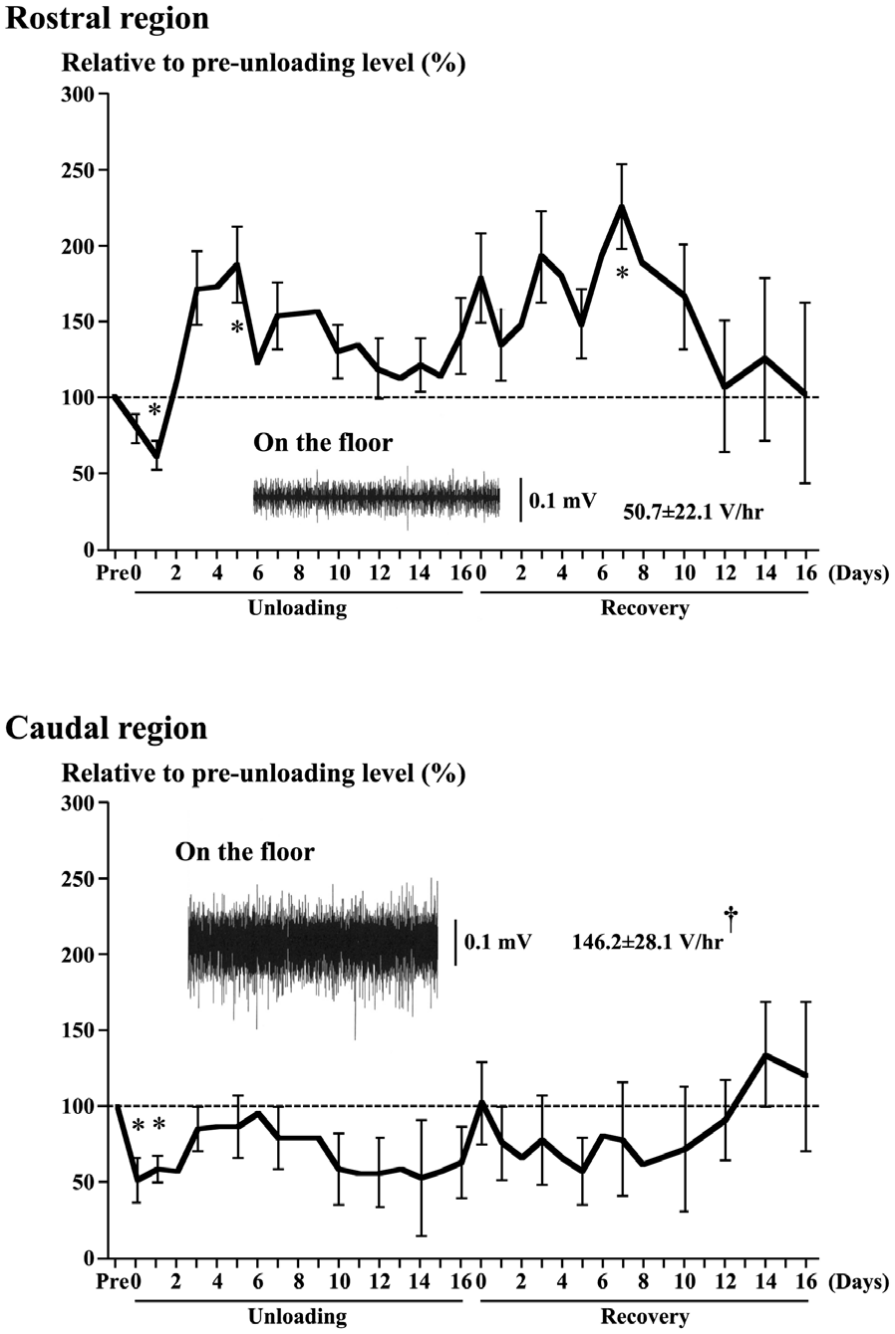
Time course changes of the mean (±SEM) electromyogram activities. Absolute activity (V/hr) and activity relative to the pre-unloading (pre) level on the floor (100%) in the rostral and caudal regions of adductor longus muscle in response to hindlimb unloading and ambulation recovery are shown. n = 5 in each region. Further, typical EMG patterns and mean activity during quadrupedal rest on the floor are also shown. *: p<0.05 vs. pre-experimental level on the floor. †: p<0.05 vs. the rostral region.

The EMG activity of AL on the floor was tonic and the integrated EMG activity level during quadrupedal resting position was greater in the caudal than the rostral region (146 vs. 51 V/hr, p<0.05, Fig. 6). The EMG activity in the caudal region decreased and became phasic following an acute unloading. The activity remained low up to day 2 (p<0.05), and increased from day 3 to 6 toward the pre-experimental level. However, it was gradually decreased again after 7th day of unloading and the mean activity at the last day of unloading was approximately 62% of the pre-suspension level (p>0.05). The activity remained low (p>0.05) even during the recovery phase, although it was increased in response to acute reloading. But, the activity level was normalized after ∼12 days. The EMG activity in the rostral region was even elevated during continuous unloading, although it was also decreased acutely (p<0.05). The mean activity tended to decrease after 6th day, but the level at the last day of 16-day unloading was still greater than the pre-experimental level on the floor (p>0.05). The activity remained high even during the ambulation recovery, but returned to the pre-experimental level after 12 days.

### Morphological responses of AL to unloading

**Figure 7:**
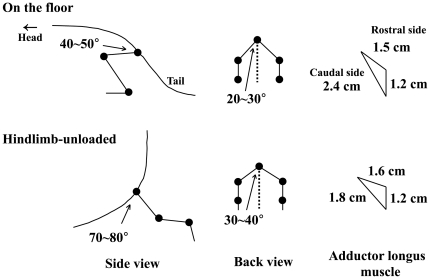
The mean angles of hip joint of rat and perimeters of adductor longus muscle. Changes of these parameters during quadrupedal resting position on the floor and hindlimb unloading are shown.

**Figure 8:**
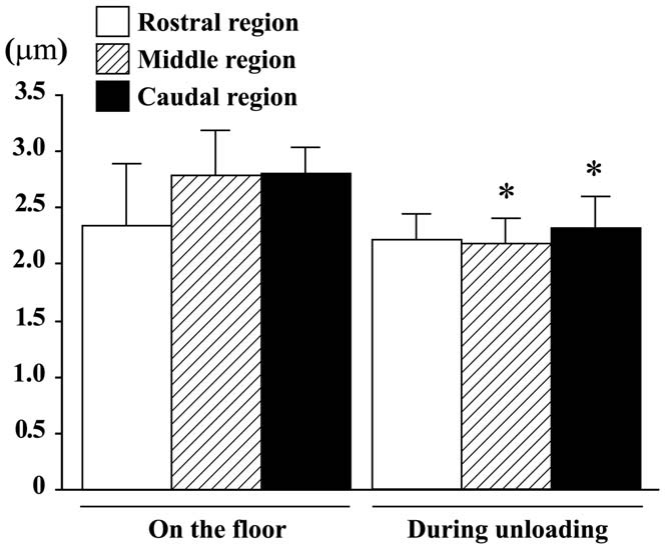
Sarcomere length of adductor longus muscle fibers. The mean levels during quadrupedal rest on the floor and hindlimb unloading are shown. Mean±SEM. n = 5 in each group/stage. *: p<0.05 vs. each region on the floor.

The mean hip joint angle from back view was approximately 20–30° and that from side view was approximately 40–50° during the sedentary quadrupedal position on the floor (Fig. 7). The hip joints were abducted to 30–40° and extended backward to 70∼80° in response to unloading. The length of fibers in the caudal region was passively shortened by gravitational unloading due to the postural change. The mean length of the outer side in the caudal region was shortened ∼25% during unloading. That in the rostral region was unchanged or even stretched slightly in response to unloading. The mean sarcomeres in the middle and caudal regions of AL, fixed keeping the angles of hip joints equivalent to those during the quadrupedal posture on the floor, tended to be slightly stretched than those in the rostral region (Fig. 8, p>0.05). The sarcomere lengths in the middle (22%) and caudal (18%), but not the rostral, regions were decreased significantly in response to unloading.

## Discussion

Responses of AL muscle fibers in Wistar Hannover rats to gravitational unloading and reloading were studied. The data suggested that the differences of basal fiber properties between the caudal and rostral regions were closely related to the neural and mechanical factors. Further, effects of unloading on the fiber properties were greater in the caudal region where the decreases of EMG activity and sarcomere length were prominent.

### Morphological properties

The unloading-related reduction of muscle weight was not directly related to the inhibited gain of body weight. The muscle weight, normalized by body weight, was significantly less than controls, suggesting that the muscle atrophy advanced regardless of the inhibited gain of body weight. The percent increases of body weight and AL weight after 16 days of ambulation were 32 and 105% vs. the weights observed immediately after the unloading, respectively. Therefore, it is clearly indicated that the rate of recovery in muscle weight was greater than that of body weight.

### Number of myonuclei and satellite cells

More number of myonuclei was distributed (∼4 times) in the fibers of caudal than the rostral region. The unloading-related fiber atrophy, which was pronounced in the caudal region especially, was not directly related to the decrease of myonuclear number, which was seen in soleus muscle [23], [24]. The level of myonuclear domain in the caudal region was decreased after unloading, but was normalized following 16 days of ambulation recovery. These results suggest that the unloading-related decrease of cytoplasmic volume was advanced, even though the number of myonuclei was not lowered, suggesting that function of myonuclei for protein synthesis may be inhibited as was reported elsewhere [23], [24].

The number of satellite cells located in fibers was ∼5 times greater in the caudal than the rostral region at the beginning of experiment. The numbers of BrdU-positive (mitotic active) satellite cells was significantly decreased by unloading in the caudal region. It was reported that significant fiber atrophy in soleus muscle of Wistar Hannover rats was directly related to the decreased number of myonuclei and satellite cells [23]. The loss of satellite cells was noted at the middle region of soleus muscle fibers sampled from tendon-to-tendon. And this phenomenon was related to the passive shortening of sarcomere length, which decreased the mechanical load. Although the tension development was not measured in the present study, the significant decrease of sarcomere length in the middle and caudal regions in response to unloading indicates that the mechanical load, applied to muscle fibers, was also decreased in these regions. The unloading-related decrease of mitotic active satellite cell number in the caudal region of AL may be related to this phenomenon. Normalization of mitotic active satellite cell number after 16-day ambulation may be closely related to the increased mechanical load as was also reported by Wang et al. [23].

### Fiber phenotype

It was reported that the shift of fiber phenotype was closely related to the turnover rate of high-energy phosphates [25]. Therefore, the prominent shift of fiber phenotype toward fast-twitch type following unloading, seen in the present study, may be caused by an inhibition of the turnover rate of high-energy phosphates, which is induced by gravitational unloading [26]. However, it is not clear why the magnitude of fiber transformation in Wistar Hannover rats was greater than that in other species, such as Czechoslovakian Wistar rats [21].

Data suggested that the basal fiber phenotype was closely related to muscle activity. At the beginning of experiment, ∼97% of fibers were pure type I in the caudal region with greater EMG activity at rest in a prone position. Meanwhile, the mean pure type I fibers in the rostral region, with less EMG activity, was only 61%. However, the shift of fiber phenotype induced by unloading was relatively uniform in all muscle regions.

### Metabolic properties

The responses of enzyme activities to unloading measured in single muscle fibers suggest that SDH activity is often maintained or even elevated in atrophied muscle fibers [21], [27], [28]. These results suggested that the unloading-related decreases of fiber CSA and enzyme level were relatively parallel or the degree of atrophy was slightly greater. Similar phenomena were observed in the current study. The magnitudes of the unloading-related loss of enzyme levels were proportional to the fiber atrophy. Although the specific activities of both SDH and GPD in single muscle fibers were not significantly different between the unloaded and cage control groups, their integrated activities, expressed ΔOD/min×fiber CSA, tended to be decreased following unloading.

### Muscle activities

#### Neural activity

The EMG activity of AL decreased instantaneously in response to hindlimb unloading, as was observed in soleus [5], [8], [10], [29]. The EMG activity in soleus muscle was gradually increased after ∼1 week toward the pre-suspension level and was normalized ∼2 weeks after unloading. However, fiber atrophy was still advanced [5], [13]. Although a similar response of EMG was also noted in AL, the initiation of increase in EMG level was noted at 3rd day of unloading, relative to 5th day in soleus [5], [8], [10]. The integrated EMG levels in the rostral and caudal region reached the peak levels (186 and 95% of the pre-suspension level) at 5th and 6th days of unloading, respectively. And these levels gradually decreased again during the rest of unloading period, as was also observed in soleus muscle [5].

But the mean EMGs in the rostral region remained higher than the pre-suspension level (p>0.05). Similarly elevated EMG activities were noted in tibialis anterior muscle, which was passively stretched due to the plantarflexion of ankle joints during continuous hindlimb suspension [5]. On the contrary, the EMG activities of ankle dorsiflexors were inhibited. Decreased EMG activity was also reported in rat soleus muscle with shortened muscle fibers and sarcomeres in response to hindlimb suspension [10]. Further, fixing of ankle joints of rats at either dorsiflexed or plantarflexed position during hindlimb suspension caused opposite responses in soleus mass [15]. Atrophy of whole muscle and fibers was completely prevented by ankle dorsiflexion, suggesting that stretching of muscle and/or application of mechanical loading prevent atrophy. But such phenomena were not seen in the muscle and fibers with plantarflexion-related inhibition of mechanical load. These results suggest that different effects on the sarcomere length and EMG activity, seen in the caudal and the rostral region, during unloading may be one of the causes for the region-specific differences in the responses of fiber properties.

#### Mechanical load

The load, applied to muscle, has been estimated by measurement of tension development of muscle by using a force transducer, placed at the distal tendon of soleus muscle and by analyzing the sarcomere length *in vivo* [10], [23]. The passive and active force production by muscle was completely inhibited due to the plantarflexion-related shortening of muscle fibers and/or sarcomeres, when the rat was hindlimb-suspended. It was also reported that the decrease of soleus EMG in response to unloading was related to the passive shortening of muscle fibers or sarcomeres, which decreases the mechanical load [10].

The sarcomeres were stretched (∼3.1 µm) in slow-twitch soleus muscle fibers of rats, when they maintained the sedentary quadrupedal posture on the floor [10], [23]. Similar phenomenon was noted in AL. The mean sarcomere length in the rostral region during the sedentary quadrupedal posture on the floor was ∼2.3 µm and that in the middle and caudal regions was ∼2.8 µm in the present study. Even though the sarcomere length in the rostral region was stable, sarcomeres in the middle and caudal regions were passively shortened to ∼2.2–2.3 µm (p<0.05) in response to acute gravitational unloading by hindlimb suspension. Our previous study showed that the passive tension development by rat soleus muscle with ∼2.2–2.3 µm sarcomere length was only 17–34% relative to that with ∼2.8 µm sarcomere length, suggesting a significant inhibition of mechanical load [10]. Although remodeling was induced and sarcomere length was slightly increased following 2 weeks of hindlimb suspension, the degree of improvement in tension development was still negligible. However, mechanical load applied to AL muscle fibers may be increased during reloading by ambulation on the floor, as was observed in soleus [23]. Responses of AL muscle to unloading and reloading were also reported elsewhere [30]–[32], as was shown below.

The time-course change of sarcomere length in AL was not determined in the present study. However, it is speculated that different sarcomere length may be maintained throughout the suspension period, since the posture of rat hindlimbs was relatively stable. Therefore, it is suggested that the significant unloading-related shortening of sarcomeres in the caudal region may be closely related to the prominent changes of fiber properties in this region.

Riley's group also investigated the responses of AL to gravitational unloading and reloading [30]–[32]. They reported fiber atrophy in response to gravitational unloading by actual spaceflight [31] and hindlimb suspension [30]. Further, sarcomere eccentric contraction-like lesions were noted in flight rat muscle 4.5 h post-flight, even though those fibers were not noted in muscle sampled in-flight [31]. But these lesions were repaired after 9 days, although regenerating fibers were still present. Eccentric contraction was also applied to AL muscle of rat following 14 days of hindlimb suspension [32]. Again, similar legions were induced in some muscle fibers. We also observed some regions with disorganization of Z-disc structure in reloaded (for 5 days) soleus muscle fibers of rats that were hindlimb-suspended for 7 days previously (unpublished observation). These results indicated that gravitational re-loading after unloading caused hyper-stretching of sarcomeres. Even though the myofibrillar damage was not checked in the present study, the data suggested an important role of mechanical stress, as well as neural activity, in the regulation of muscle fiber properties.

Kawano et al. [24] reported that unloading-related fiber atrophy (−52%) in rat soleus muscle was closely associated with the decreased levels of phosphorylated ribosomal protein S6 (S6, −98%) and 27-kDa heat shock protein (HSP27, −63%). On the contrary, increased levels of phosphorylated S6 (+84%) and HSP27 (+28%) were seen in fibers with hypertrophy (+24%), caused by ablation of synergists. However, Sugiura et al. [33] reported that the levels of phosphorylated S6 and p70^S6K^ in soleus muscle did not change significantly after 10-day hindlimb unloading of rats.

It was further reported that the level of phosphorylated protein kinase B, known as Akt, was decreased in atrophied soleus muscle caused by unloading [33]. Some papers suggested that unloading-caused atrophy was related to the degradation of contractile proteins through ubiquitin-dependent proteolytic pathway [24], [34]. Early activation of Ca^2+^-dependent proteases (calpains) was also reported as one of the factors for induction of unloading-related muscle atrophy [35]–[37]. However, the signaling pathway in the region-specific responses of AL muscle to unloading and reloading is still unclear, even though it was suggested that both neural and mechanical factors play important roles in the present study.

### Conclusion

Effects of gravitational unloading and reloading on the properties of AL muscle were studied in rats. The caudal, not the rostral, region was mainly composed of fibers expressing pure type I MHC. More numbers of myonuclei and satellite cells were seen in the caudal than rostral region. These properties were closely related to the greater levels of EMG and of sarcomere length. Significant decrease of mitotic active satellite cells and myonuclear domain, and inhibition of growth-associated increase of myonuclear number were seen only in the caudal region. Such responses of fiber properties were also closely related to the inhibition of the EMG activities and passive shortening of sarcomeres. Although the fiber type changes were relatively uniform across all muscle regions, the regional changes related to fiber size, the numbers of myonuclei, and satellite cell activation were consistent with the different activation and length of muscle fibers or sarcomeres during the adaptation period. These results suggest that the region-specific properties of AL muscle fibers are closely related to both neural and mechanical factors.

## Supporting Information

Figure S1 Fiber-type-specific activity of succinate dehydrogenase in whole fibers of adductor longus muscle. Mean±SEM. n = 5 in each group/stage. Pre, Day 16, and Day 32: Before unloading, immediately after termination of unloading or cage housing, and 16 days after ambulation recovery on the floor or 32 days of cage housing, respectively. I, II, and I+II: Fibers expressing pure type I (slow) and II (fast), and co-expressing both type I and II myosin heavy chain, respectively. OD: optical density.
(TIF)

Figure S2 Fiber-type-specific integrated activity of succinate dehydrogenase in whole fibers of adductor longus muscle. Mean±SEM. n = 5 in each group/stage. See Figure S1 for the abbreviations.
(TIF)

Figure S3 Fiber-type-specific activity of α-glycerophosphate dehydrogenase in whole fibers of adductor longus muscle. Mean±SEM. n = 5 in each group/stage. See Figure S1 for the abbreviations.
(TIF)

Figure S4 Fiber-type-specific integrated activity of α-glycerophosphate dehydrogenase in whole fibers of adductor longus muscle. Mean±SEM. n = 5 in each group/stage. See Figure S1 for the abbreviations.
(TIF)

Materials S1 (DOC)
