# Supplementary figures and images for: Region-Specific Responses of Adductor Longus Muscle to Gravitational Load-Dependent Activity in Wistar Hannover Rats
Source: PLoS One. 2011 Jun 22;6(6):e21044. doi: 10.1371/journal.pone.0021044 (PMC3120817; doi:10.1371/journal.pone.0021044)

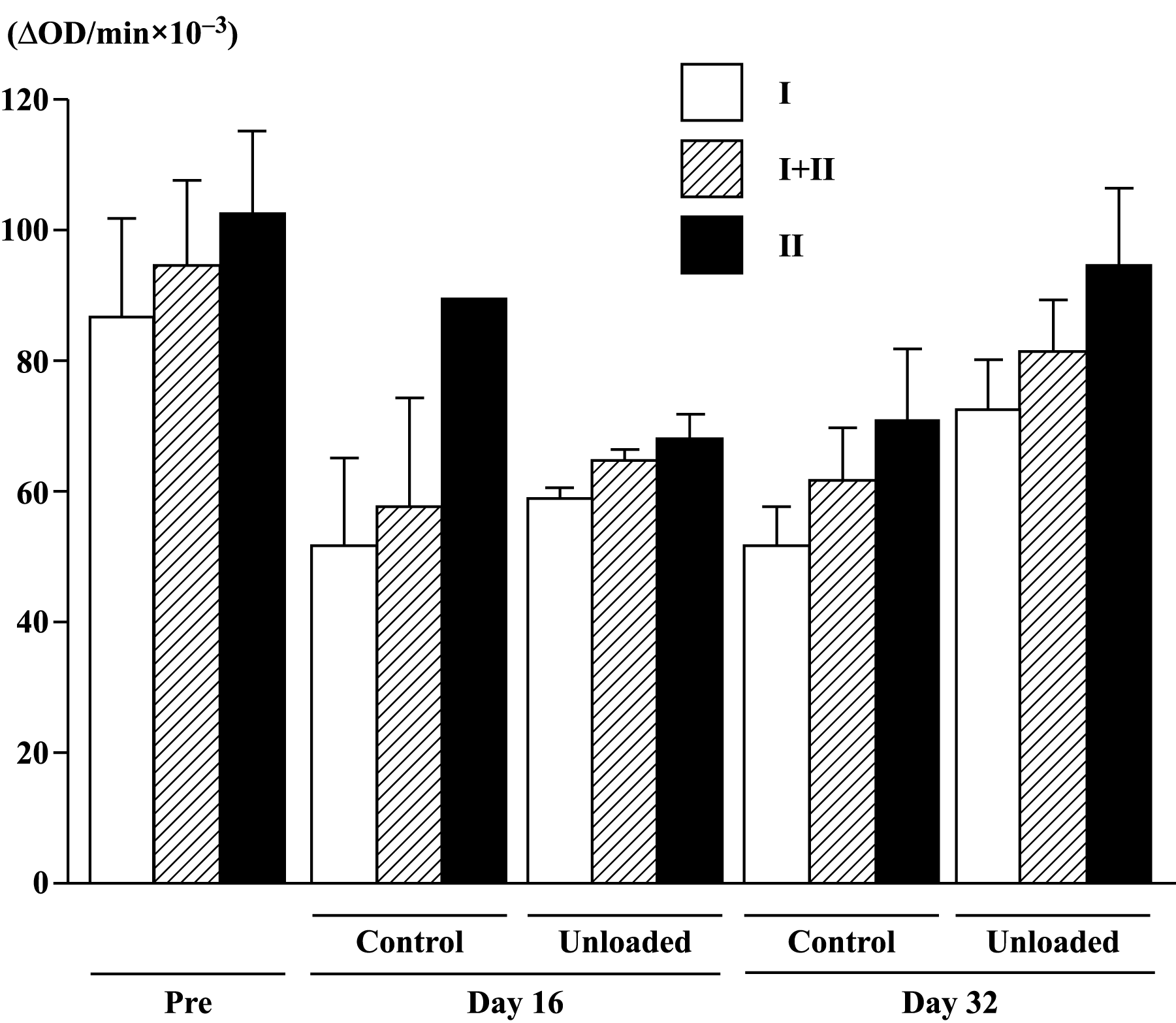

Supplement: Figure S1 — Fiber-type-specific activity of succinate dehydrogenase in whole fibers of adductor longus muscle. Mean±SEM. n = 5 in each group/stage. Pre, Day 16, and Day 32: Before unloading, immediately after termination of unloading or cage housing, and 16 days after ambulation recovery on the floor or 32 days of cage housing, respectively. I, II, and I+II: Fibers expressing pure type I (slow) and II (fast), and co-expressing both type I and II myosin heavy chain, respectively. OD: optical density. (TIF) [file pone.0021044.s001.tif]

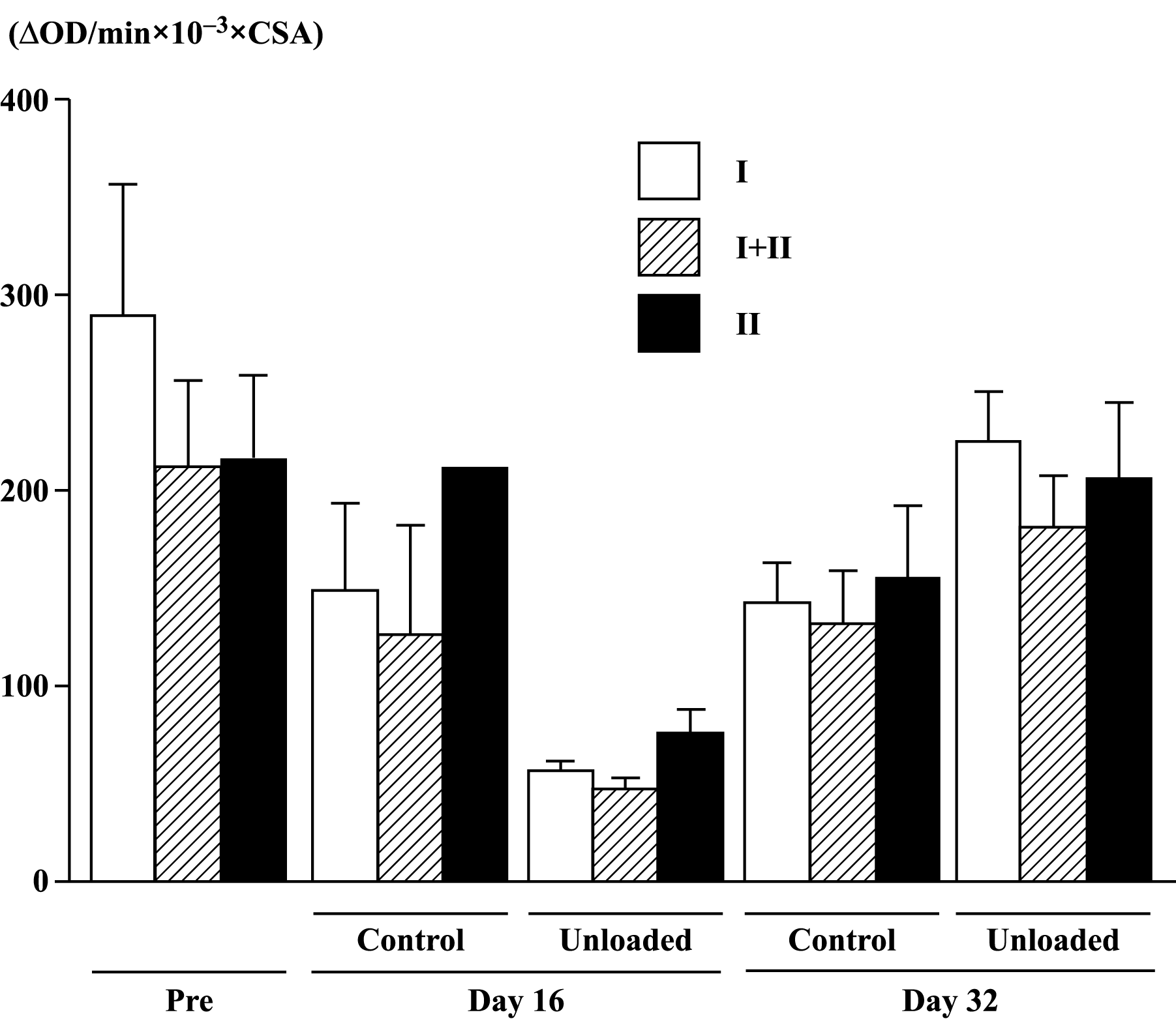

Supplement: Figure S2 — Fiber-type-specific integrated activity of succinate dehydrogenase in whole fibers of adductor longus muscle. Mean±SEM. n = 5 in each group/stage. See Figure S1 for the abbreviations. (TIF) [file pone.0021044.s002.tif]

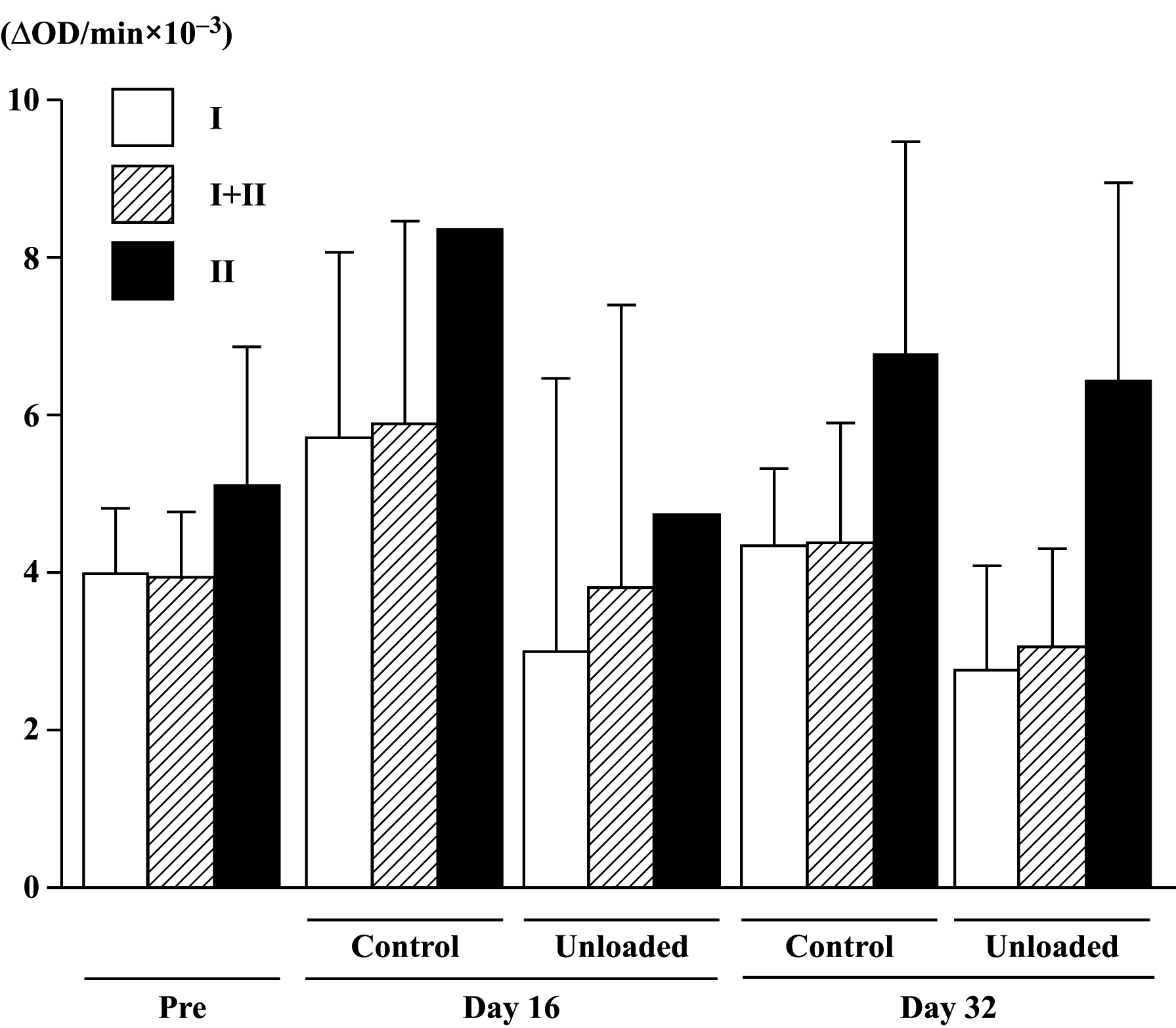

Supplement: Figure S3 — Fiber-type-specific activity of α-glycerophosphate dehydrogenase in whole fibers of adductor longus muscle. Mean±SEM. n = 5 in each group/stage. See Figure S1 for the abbreviations. (TIF) [file pone.0021044.s003.tif]

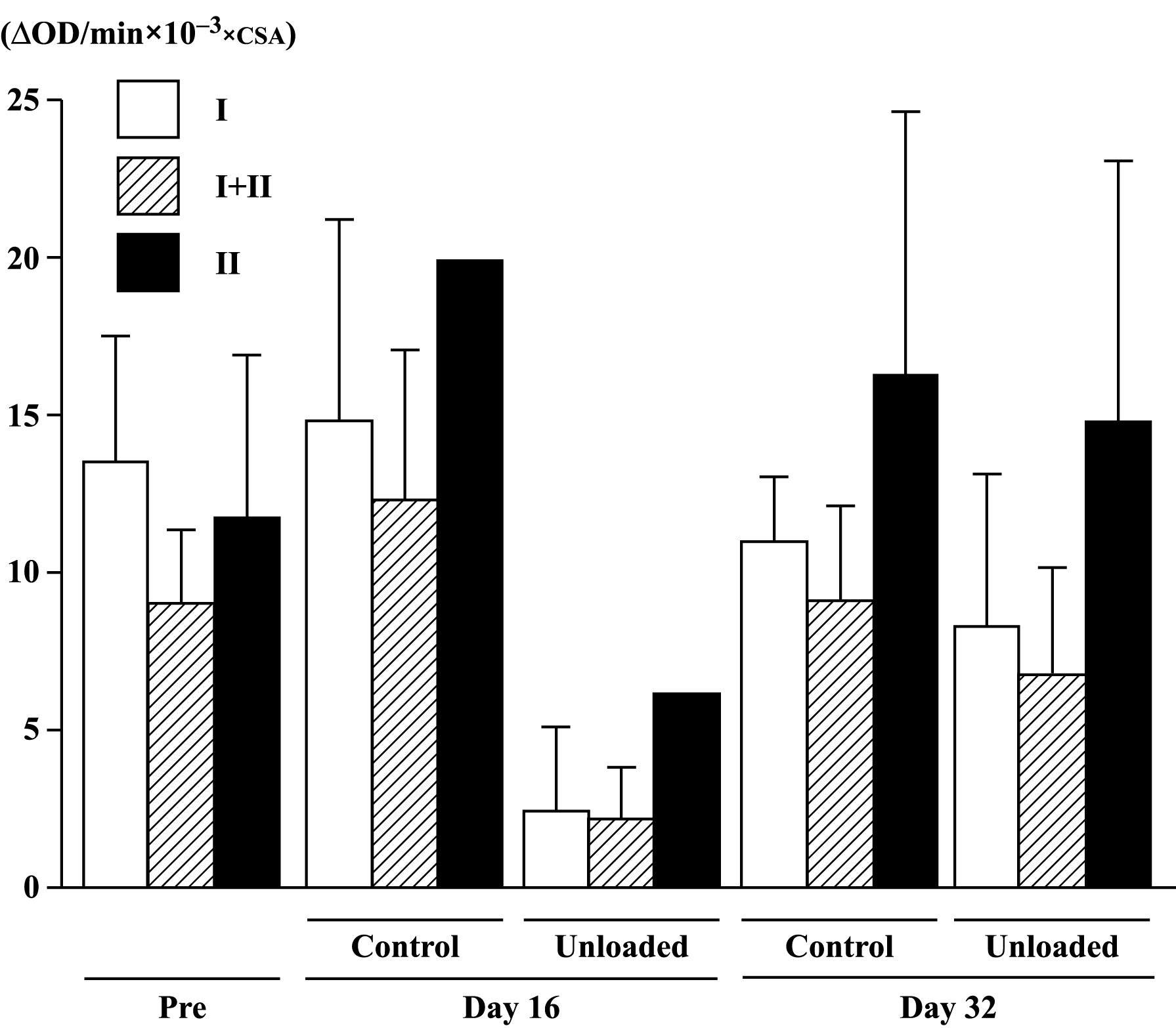

Supplement: Figure S4 — Fiber-type-specific integrated activity of α-glycerophosphate dehydrogenase in whole fibers of adductor longus muscle. Mean±SEM. n = 5 in each group/stage. See Figure S1 for the abbreviations. (TIF) [file pone.0021044.s004.tif]
