# Supplementary material for: Region-Specific Responses of Adductor Longus Muscle to Gravitational Load-Dependent Activity in Wistar Hannover Rats
Source: PLoS One. 2011 Jun 22;6(6):e21044. doi: 10.1371/journal.pone.0021044 (PMC3120817; doi:10.1371/journal.pone.0021044)
Supplement: Materials S1 — (DOC) [file pone.0021044.s005.doc]

**SUPPLEMENTAL MATERIALS**

**Methods**

**Enzyme activities in fiber cross-section**

Quantitative analyses of the activities of succinate dehydrogenase and α-glycerophosphate dehydrogenase were determined in adductor longus (AL) muscle histochemically, as was described elsewhere [1, 2]. The images of the same area of each section were incorporated into a computer (Power Mac G3, Apple). The stained images were digitized as grey-level pictures using Scion image [2, 3]. Arrays of picture elements were quantified to 256 grey levels that were then converted automatically to an optical density (OD). The fiber cross-sectional areas (CSAs) were also analyzed. The fibers were matched with those analyzed for fiber phenotype. Each fiber stained with or without substrate was also matched. Sections incubated without the substrate were used as tissue blanks to correct for non-specific staining that occurred during the reaction. The level of OD in the section incubated without substrate was subtracted from the level in the section stained with substrate. The final OD level was divided by the incubation time and the enzyme activities were expressed as the specific activity (ΔOD/min) and integrated activities (ΔOD/min × fiber CSA). The OD level of each fiber was matched with that of fiber analyzed the myosin heavy chain expression in the serial sections.

**Recording of electromyogram**

Responses of electromyogram activities in AL muscle to hindlimb unloading were determined in 5 male Wistar Hannover rats (5 weeks old). For implantation of electrodes, a skin incision in rat, anesthetized with *i.p.* injection of sodium pentobarbital (5 mg/100g body weight), was made along the sagittal suture of the skull after shaving and cleaning with betadine. The exposed skull was dried and a head plug connector was firmly anchored to the skull using both screws and dental cement. Five enamel-coated constantan wires with 80 µm diameter were led subcutaneously from the connector to the back region.

The left AL muscle was exposed keeping the blood and nerve supplies intact. Bipolar electrodes were implanted into the caudal and rostral portion of AL. The wires were inserted by threading individually through a 27-gauge hypodermic needle being passed through the muscle individually. The needle was carefully withdrawn and the insulated wire was stripped (~1 mm). The section of the wire, with the insulation removed, was implanted into the mid-belly region of the muscle. Two wires were inserted in parallel with the muscle fibers (~2 mm apart). A wire for the common ground was also implanted at the back region. Each wire was secured with a suture at its entry and exit from the muscle, so that the stripped portion of the wire in the muscle was fixed. Then, the skin was sutured. The exposed portions of wires at the head plug were connected to the recorder (PC216AX, Sony, Tokyo) using flexible insulated cables after 2 days of recovery from the surgery.

**References**

1. Jiang B, Ohira Y, Roy RR, Nguyen EI, Ilyina-Kakueva E, et al. **(**1992**)** Adaptation of fibers in fast-twitch muscles of rats to spaceflight and hindlimb suspension. J Appl Physiol 73: 58S-65S.
2. Ohira Y, Jiang B, Roy RR, Oganov V, Ilyina-Kakueva E, et al. **(**1992**)** Rat soleus muscle fiber responses to 14 days of spaceflight and hindlimb suspension. J Appl Physiol 73: 51S-57S.
3. Martin TP, Vailas AC, Durivage JB, Edgerton VR, Castleman KR **(**1985**)** Quantitative histochemical determination of muscle enzymes. J Histochem Cytochem 33: 1053-1059.
